# Targeting Mitochondrial Vulnerabilities in Chronic Myeloid Leukemia: From Pathobiology to Novel Therapeutic Opportunities

**DOI:** 10.3390/cancers18060982

**Published:** 2026-03-18

**Authors:** Francesco Caprino, Ilenia Valentino, Antonella Bruzzese, Ludovica Ganino, Maria Mesuraca, Rita Citraro, Massimo Gentile, Maria Eugenia Gallo Cantafio, Nicola Amodio

**Affiliations:** 1 Department of Experimental and Clinical Medicine, Magna Graecia University of Catanzaro, 88100 Catanzaro, Italy; f.caprino@unicz.it (F.C.); ilenia.valentino@studenti.unicz.it (I.V.); ludovica.ganino@studenti.unicz.it (L.G.); mes@unicz.it (M.M.); 2 Hematology Unit, Department of Onco-Hematology, Azienda Ospedaliera Annunziata, 87100 Cosenza, Italy; a.bruzzese@aocs.it (A.B.); massimo.gentile@unical.it (M.G.); 3 Department of Science of Health, Magna Graecia University of Catanzaro, 88100 Catanzaro, Italy; citraro@unicz.it; 4 Department of Pharmacy, Health and Nutritional Sciences, University of Calabria, 87036 Rende, Italy

**Keywords:** Chronic Myeloid Leukemia, metabolic reprogramming, mitochondrial dysfunction, oxidative stress

## Abstract

**Background**: Mitochondria are multifunctional organelles that play a central role in maintaining cellular homeostasis by regulating energy metabolism, reactive oxygen species (ROS) generation, ion homeostasis, and apoptotic signaling. Dynamic processes such as mitochondrial fission, fusion, and intracellular trafficking enable cells to adapt to metabolic and environmental stress. Growing evidence indicates that dysregulation of these processes is a hallmark of cancer, contributing to metabolic reprogramming, redox imbalance, evasion of apoptosis, and disease progression. This narrative review aims to discuss the role of mitochondrial alterations in the pathophysiology of chronic myeloid leukemia (CML) and their potential therapeutic implications. **Methods**: Original research articles published between 2010 and 2025 were considered in this narrative review. The selected studies were critically discussed and categorized into three principal thematic domains: mitochondrial regulation of redox homeostasis, metabolic rewiring, and control of cell death pathways. Evidence was synthesized to elucidate the contribution of mitochondrial dysfunction to CML initiation, progression, and therapeutic resistance. **Results**: The reviewed studies highlight how mitochondrial abnormalities play a pivotal role in BCR-ABL1-driven leukemogenesis. Alterations in mitochondrial metabolism and ROS signaling support sustained proliferative signaling, promote genomic instability, and facilitate resistance to apoptosis. In addition, mitochondrial adaptations contribute to resistance to tyrosine kinase inhibitors (TKIs) and are essential for the persistence and survival of leukemic stem cells. **Conclusions**: Mitochondria emerge as central regulators of CML pathobiology. Therapeutic strategies targeting mitochondrial metabolism, redox homeostasis, and apoptotic signaling pathways represent promising approaches to overcoming TKI resistance and may improve clinical outcomes for patients with CML.

## Simple Summary

Chronic myeloid leukemia (CML) is a blood cancer characterized by metabolic defects, including dysregulated energy management, impaired redox balance, and resistance to programmed cell death. Central to these processes are mitochondria, as essential regulators of cellular energy production, survival, and apoptosis. In CML, mitochondrial dysfunction supports disease progression and contributes to therapeutic resistance. This review highlights the role of altered mitochondrial biology in CML pathogenesis and explores potential therapeutic strategies that target mitochondrial function as a means to enhance treatment efficacy and overcome drug resistance.

## 1. Introduction

Chronic myeloid leukemia (CML) is a clonal hematopoietic stem cell malignancy characterized by uncontrolled proliferation of the granulocytic lineage and, if untreated, progression to acute leukemia. It is a relatively uncommon disease with a median age at diagnosis of approximately 65 years and a male predominance. Clinical presentation is often nonspecific, including fatigue, anorexia, and weight loss, although up to 40% of patients are asymptomatic at diagnosis. In such cases, CML is frequently detected incidentally through routine laboratory testing demonstrating leukocytosis. Splenomegaly is the most common physical finding, present in approximately half of patients at initial presentation [1,2,3]. The pathogenesis of CML is primarily driven by the chromosomal translocation t(9;22)(q34;q11), which results in the formation of a shortened chromosome 22, known as the Philadelphia (Ph) chromosome. This translocation juxtaposes the ABL1 gene from chromosome 9 with the BCR gene on chromosome 22, leading to the formation of a BCR-ABL1 fusion gene. The resulting chimeric gene encodes a fusion protein with constitutively active tyrosine kinase activity. The breakpoint in the ABL1 gene typically occurs within a 300-kb intronic region between exons 1a, 1b, and 2. In contrast, breakpoints in the BCR gene can occur at multiple sites, giving rise to at least three distinct fusion transcripts that encode proteins of different molecular weights and tyrosine kinase activity:

• p190, that results from a breakpoint between BCR exons e1–e2 (minor breakpoint cluster region, m-bcr), commonly associated with acute lymphoblastic leukemia (ALL).
• p210, that arises from a breakpoint within exons b1–b5 (major breakpoint cluster region, M-bcr, ~5.8 kb), and is the most frequent transcript in CML.
• p230, that involves a breakpoint between exons e19–e20 (centromeric to M-bcr), and is typically seen in a subset of chronic neutrophilic leukemia (CNL) cases [4].

BCR-ABL1 fusion protein is aberrantly localized to the cytoplasm, where it undergoes autophosphorylation at multiple tyrosine residues, becoming constitutively active. Structural changes in certain BCR domains further facilitate interactions with various signaling proteins, activating multiple downstream pathways. Collectively, these alterations endow the BCR-ABL1 fusion protein with potent leukemogenic capacity through several key mechanisms:

• Impaired adhesion: the protein alters the interaction of myeloid progenitors with the bone marrow stroma and extracellular matrix, enhancing their egress into peripheral blood and reducing regulatory signals from the microenvironment.
• Mitogenic signaling activation: it activates proliferative pathways—primarily the RAS/MAPK axis—via both direct phosphorylation and intermediary molecules such as Shc and Crkl.
• Genomic instability: accelerated proliferation impairs DNA repair mechanisms at the G1/S checkpoint, promoting the accumulation of additional mutations and chromosomal aberrations, which drive progression to the accelerated or blastic phase.
• Apoptosis inhibition: the BCR-ABL1 protein interferes with programmed cell death through incompletely understood mechanisms, although its inhibition has been shown to restore apoptotic responses.
• Disruption of HSC homeostasis: this results in the expansion of a leukemic clone with altered self-renewal and differentiation properties.

Initially, Ph-positive HSCs retain partial differentiation capacity, producing the granulocytic hyperplasia characteristic of the chronic phase. However, with time, additional genetic lesions accumulate, progressively impairing differentiation and driving transformation to the accelerated or blast phase [5,6,7,8,9,10,11,12,13,14].

The most consistent indicators of disease progression include the emergence of additional cytogenetic abnormalities (ACAs) and increased blast counts. The most frequently observed ACAs in blast crisis were designated as the “major route” by Mitelman et al., and include trisomy 8 (+8), duplication of the Ph chromosome (+Ph), isochromosome 17q [i(17q)], and trisomy 19 (+19) [15,16]. These abnormalities are associated with poor prognosis, whether present at diagnosis or acquired during treatment [17,18]. Other cytogenetic alterations associated with adverse outcomes include rearrangements involving 3q26.2 and 11q23, and monosomy 7 or 7q deletion (−7/7q−) [19,20]. In the CML Study IV, Helman et al. analyzed outcomes in 1510 patients treated with imatinib. Based on their prognostic impact, ACAs were stratified into high-risk ACAs: +8, +Ph, i(17q), +17, +19, +21, 3q26.2, 11q23, −7/7q−, or complex karyotypes; or low-risk ACAs, including all other abnormalities [21].

### 1.1. CML Stem Cells

CML is characterized by a hierarchical architecture of malignant cells, with a small population of leukemic stem cells (LSCs) occupying the top tier. These LSCs possess fundamental properties similar to normal HSCs, such as the ability to self-renew, differentiate into multiple blood cell lineages, and maintain a largely dormant or quiescent state. However, LSCs are uniquely defined by oncogenic reprogramming primarily driven by the BCR-ABL fusion protein, along with additional genetic, epigenetic, and transcriptional modifications that promote their survival and enable resistance to standard treatments [22,23]. These alterations involve enhanced expression of genes related to stem cell maintenance and survival, improved DNA damage repair mechanisms, and epigenetic remodeling mediated by factors such as PRC2, histone deacetylases, and DNA methyltransferases [22,24,25].

These molecular alterations prompt LSC proliferation, enable evasion of apoptosis, and allow adaptation to environmental and therapeutic stressors. Notably, despite effective inhibition of BCR-ABL kinase activity by TKIs, LSCs activate alternative signaling and metabolic pathways—including PI3K/AKT, JAK/STAT, and hypoxia-inducible factor-1 α (HIF-1α)—that promote stem cell persistence and therapeutic resistance [22,23], contributing to minimal residual disease and eventual relapse [26].

### 1.2. CML Therapeutic Options

In Western countries, life expectancy of newly diagnosed patients with chronic phase (CP) CML now approaches that of age-matched controls, largely due to the use of TKIs [15,27,28,29,30,31]. Given the success of imatinib, several TKIs have been developed and approved for frontline therapy. Treatment choice depends on efficacy, safety, cost, and increasingly on quality of life and long-term toxicity. A major therapeutic goal is the achievement of treatment-free remission (TFR) [15].

The IRIS trial first demonstrated imatinib’s superiority over interferon-α (IFN-α) plus cytarabine in newly diagnosed CP-CML. With a median follow-up of 19 months, complete cytogenetic response (CCyR) rates were 74% with imatinib versus 9% with IFN-α (*p* < 0.001). Major molecular responses (MMR) were also more frequent with imatinib (39% vs. 2%) [32].

To overcome resistance, second-generation TKIs (2G-TKIs) such as dasatinib, nilotinib, and bosutinib were developed. Dasatinib, with 350-fold greater potency than imatinib, showed higher 12-month CCyR (77% vs. 66%, *p* < 0.007) and faster molecular response in the DASISION trial [33]. In ENESTnd, nilotinib at both 300 and 400 mg twice daily achieved higher MMR at 12 months (44% vs. 22%) and higher CCyR at 24 months (87–85% vs. 77%) compared to imatinib [34].

According to the European LeukemiaNet (ELN) guidelines, imatinib, dasatinib, and nilotinib are all recommended first-line treatment options. Treatment selection should consider patient age, comorbidities, and the likelihood of achieving TFR. 2G-TKIs achieve deep molecular responses (DMR) more frequently, making them a preferred option for younger patients [15].

TKI intolerance is the most common reason for treatment switching. Toxicities vary widely and may affect quality of life. Hematologic adverse events (AEs) are generally mild and transient, whereas non-hematologic AEs—such as pleural effusion with dasatinib or pancreatitis with nilotinib—can be more severe [15,33,34].

In cases of resistance, mutational testing is essential to guide the selection of the most appropriate TKI [21]. In contrast, switching therapy because of intolerance is guided by physician judgment, patient preference, and the depth of treatment response [15]. The ELN guidelines do not specify which 2G-TKI should be selected after intolerance; however, clinical data support the use of dasatinib, nilotinib, and bosutinib [35,36,37,38,39,40]. Ponatinib has also shown efficacy in this setting [41]. In case of TKI failure, disease restaging and mutational analysis using Sanger sequencing or next-generation sequencing (NGS) are recommended. Specific mutations guide TKI selection: nilotinib for V299L, F317L; dasatinib/bosutinib for Y253H, E255K/V, F359C/V; and ponatinib or asciminib for T315I. In the absence of detectable mutations, treatment choice should be based on comorbidities, toxicity profile, and cost considerations [42,43,44,45]. Switching between 2G-TKIs after treatment failure is generally discouraged; instead, third-generation TKIs (ponatinib or asciminib) or allogeneic stem cell transplantation (allo-SCT) should be considered in eligible patients [15].

Ponatinib treatment should start at 45 mg/day in patients with T315I mutations or advanced disease, and at lower starting doses in other patients. Rigorous management of cardiovascular risk is essential. The benefit of prophylactic aspirin or anticoagulation remains uncertain [15,46,47].

Asciminib, an allosteric inhibitor targeting the BCR-ABL1 myristoyl site, showed superior major molecular response (MMR) at 6 months compared with bosutinib (25.5% vs. 13.2%, *p* = 0.029), with fewer grade ≥3 AE in the ASCEMBL trial [48]. It is approved for patients who have failed ≥2 prior TKIs at a dose of 40 mg twice a day, and at 200 mg twice a day for patients harboring the T315I mutation.

In a recent phase III trial, asciminib achieved higher 12-month MMR rate compared to imatinib (69.3% vs. 40.2%, *p* < 0.001) and numerically higher rates than 2G-TKIs (66% vs. 57.8%) [48]. Direct comparisons with ponatinib remain limited. Available analyses suggest higher MMR rates with ponatinib, whereas asciminib may confer better failure-free survival (FFS), particularly in patients without the T315I mutation [49,50].

## 2. Mitochondrial Dysregulation in CML

Mitochondria are organelles commonly referred to as the “powerhouses” of the cell because of their central role in energy production, but they also perform a wide range of additional functions including the generation of ROS, regulation of ion homeostasis, and control of biosynthetic and apoptotic pathways [51]. Mitochondria continuously undergo dynamic processes such as fission (fragmentation), fusion (merging), and intracellular trafficking [52]. These diverse functions make mitochondria key cellular sensors that enable adaptation to environmental conditions and support growth, survival, and malignant transformation. Dysregulation of mitochondrial dynamics contributes to metabolic reprogramming, cell cycle progression, and the regulation of cell death pathways—mechanisms increasingly recognized as critical in the pathogenesis of multiple malignancies [53], including both solid tumors [54,55], and hematological malignancies [56,57,58]. Herein, we analyzed the contribution of mitochondrial abnormalities to the pathobiology of CML and their relevance as potential targets for therapeutic intervention. The reviewed papers were primarily categorized into three key areas: redox homeostasis, metabolic reprogramming, and the regulation of cell death pathways.

### 2.1. Mitochondrial Regulation of Oxidative Stress and DNA Damage

**Figure 1:**
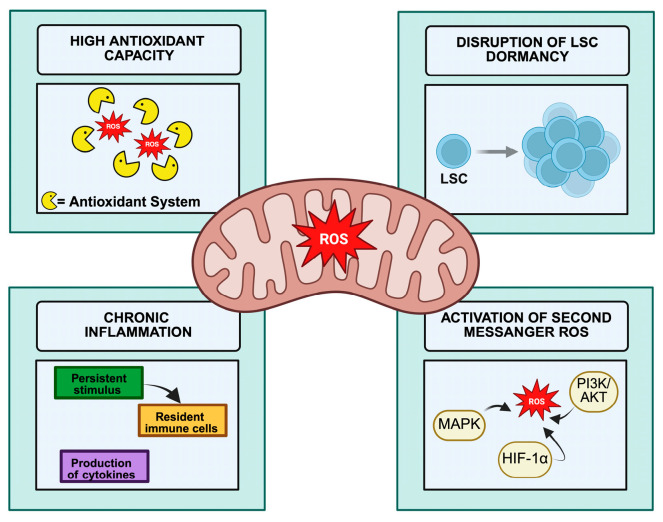
Mitochondrial Regulation of ROS and Signaling Pathways in CML. This figure illustrates the key processes involved in oxidative stress in CML. High antioxidant capacity reduces ROS levels, disrupts LSC dormancy, and promotes entry into the cell cycle. Conversely, chronic inflammation further amplifies ROS production, resulting in persistently elevated basal ROS levels. Excessive ROS act as secondary messengers that activate tumor-promoting signaling pathways, including MAPK, PI3K/AKT, and HIF-1α, thereby supporting leukemic cell survival, proliferation, and disease progression. Created with BioRender.com.

During OXPHOS, mitochondria represent a major intracellular source of ROS, primarily generated by electron leakage from complexes I and III of the electron transport chain (ETC). In cancer cells, elevated metabolic activity and mitochondrial remodeling often result in elevated basal ROS levels [59]. At moderate concentrations, ROS function as secondary messengers that activate tumor-promoting signaling pathways, while elevated ROS levels may result in cytotoxicity [60]. The impact of ROS-dependent oxidative stress in CML is graphically summarized in Figure 1.

#### 2.1.1. Mitochondrial ROS as Signaling Mediators in BCR-ABL Leukemogenesis

In CML, ROS exert highly context-dependent functions, particularly in the regulation of LSCs behavior and therapeutic responsiveness. Preclinical studies using murine models and patient-derived CML cells demonstrate that several therapeutic strategies increase ROS production from either mitochondrial or cytoplasmic sources, resulting in cytotoxic stress. This effect is especially evident in combination regimens; for example, dual inhibition of HIF-1 and TK increases ROS levels, disrupts metabolic dormancy, and drives quiescent LSCs into cell cycle entry, thereby reducing their long-term regenerative capacity. In these settings, ROS primarily function as metabolic and signaling mediators [22,23].

Mechanistically, ROS homeostasis in CML is finely regulated by enzymatic sources such as NADPH oxidases. NOX2 overactivation contributes to excessive ROS production and mitochondrial stress; however, genetic silencing of NOX2 paradoxically increases mitochondrial ROS through compensatory NOX4 activity, leading to redox imbalance [26].

Oncogenic BCR-ABL signaling further reshapes redox control by suppressing thioredoxin-interacting protein (TXNIP), a tumor suppressor regulating oxidative stress and metabolic regulation. TXNIP downregulation increases ROS production and supports leukemic cell survival, whereas restoration of TXNIP expression promotes oxidative stress-dependent cell death and slows disease progression in vivo [61].

ROS generation is closely linked to therapeutic response. Imatinib-sensitive CML cells accumulate higher ROS levels and display increased DNA damage after treatment, whereas resistant cells exhibit enhanced antioxidant capacity, including elevated glutathione peroxidase (GPX), catalase, and glutathione (GSH), which collectively protect against oxidative injury and promote drug resistance [62]. Persistent CML cells surviving imatinib exposure show increased mitochondrial mass and sustained mitochondrial ROS production, consistent with a state of chronic oxidative stress which partially occurs in a STAT3-dependent fashion [63,64]. Additional perturbations in mitochondrial quality control, i.e., the coordinated mechanisms that preserve mitochondrial integrity and function (including antioxidant defense systems, mitophagy, and mitochondrial biogenesis) also disrupt redox balance [53]. Inhibition of autophagy impairs mitophagy, leading to accumulation of dysfunctional mitochondria and increased ROS production, which promotes leukemic cell differentiation and alters cell fate decisions [65]. Similarly, pharmacological inhibition of dihydroorotate dehydrogenase (DHODH), a mitochondrial enzyme involved in pyrimidine biosynthesis, increases ROS levels, disrupts mitochondrial membrane potential, and induces CML cytotoxicity [66].

#### 2.1.2. ROS-Dependent DNA Damage and Checkpoint Modulation

When ROS exceed cellular buffering capacity, oxidative damage accumulates in macromolecules. To counteract this threat, malignant cells frequently enhance antioxidant defenses, including mitochondrial manganese superoxide dismutase (MnSOD), GPX and the thioredoxin system [67]. Tight regulation of this redox balance is critical for LSC maintenance. In this context, the hematopoietic microRNA-142 (miR-142) emerges as a key regulator of oxidative stress responses and mitochondrial metabolism. In primary CML cells, miR-142 deficiency enhances mitochondrial OXPHOS while maintaining low intracellular ROS levels through activation of the antioxidant transcription factor NRF2, a direct miR-142 target. NRF2-driven antioxidant gene expression preserves redox homeostasis, limits oxidative DNA damage, and promotes LSC survival, thereby contributing to disease progression toward blast crisis [24]. Similarly, deletion of SIRT1, a NAD^+^-dependent deacetylase involved in stress adaptation and metabolic regulation, disrupts stem cell quiescence and increases sensitivity to stress without directly inducing DNA damage, highlighting the contribution of metabolic activation to redox vulnerability [25].

Direct genotoxic effects of ROS have been demonstrated using several pharmacological models. Taxodione, a redox-active diterpenoid natural product, induces robust ROS elevation in BCR-ABL-positive cells, leading to DNA fragmentation reverted by antioxidant treatment, thereby establishing a causal link between ROS and genotoxicity [68]. Likewise, Amsacrine, a synthetic DNA-intercalating topoisomerase II inhibitor used as an anticancer agent, promotes oxidative DNA damage through NOX4-dependent ROS production mediated by the SIDT2/NOX4/ERK/HuR axis, independently of mitochondrial ROS amplification [69].

Ionizing radiation further supports the link between oxidative stress and DNA damage. Low-dose radiation induces modest apoptosis but elicits time-dependent mitochondrial dysfunction and DNA damage, consistent with sustained oxidative stress [70]. Similarly, ultraviolet (UV) exposure triggers stronger oxidative DNA damage and apoptotic signaling in imatinib-resistant BCR-ABL1-positive cells compared with sensitive counterparts, reflecting altered redox adaptability [71]. Persistent oxidative stress in LSCs harboring a folate receptor 3 (FOLR3) single-nucleotide polymorphism (SNP) has also been linked to enhanced clonogenic capacity, replicative senescence, and increased inflammatory signaling, suggesting long-term biological consequences of chronic ROS elevation [72].

Metabolic perturbations may further integrate oxidative stress with genomic instability. Loss of AMD1, a rate-limiting enzyme in polyamine synthesis, reduces polyamine levels, increases mitochondrial ROS, and promotes differentiation of LSCs, effects reversible by spermidine supplementation [73]. Elevated ROS in CML neutrophils disrupt NF-κB signaling via S-glutathionylation and alter inflammatory pathways such as iNOS expression [74]. Chronic inflammation amplifies oxidative stress, promoting DNA mutations and strand breaks and establishing a feed-forward loop of genomic damage and disease persistence [75].

Despite the presence of DNA damage, oncogenic BCR-ABL signaling can attenuate it by interfering with p53 post-translational regulation and activity, thereby overriding canonical DNA damage checkpoints [76]. Conversely, pharmacological disruption of 14-3-3 protein interactions, which keep BCR-ABL into the cytoplasm, restores c-Abl activity and reactivates apoptosis downstream of DNA damage, sensitizing both imatinib-sensitive and -resistant CML cells to cell death [77].

Perturbation of microenvironmental interactions further influences mitochondrial redox homeostasis. Disruption of CXCR4-dependent stromal protection indirectly alters mitochondrial function and sensitizes CML cells to stress-induced death [78]. Moreover, oxidative stress may extend beyond leukemic cells to the microenvironment, as dasatinib increases intracellular and mitochondrial ROS and induces oxidative DNA lesions, such as 8-oxo-dG, in endothelial cells, contributing to vascular damage [79].

#### 2.1.3. Pharmacological Exploitation of Redox Vulnerability

A broad range of natural and synthetic compounds endowed with anti-tumor activity further highlight the contribution of ROS to leukemic cytotoxicity [80,81].

Natural products and naturally derived compounds exhibit broad antitumor activity in both solid tumors [82] and hematological malignancies [56,83,84,85,86] through complementary mechanisms, including inhibition of histone deacetylases, modulation of proteasome activity, and suppression of key survival and epigenetic pathways. Among these, Glaucocalyxin A (GLA), a bioactive diterpenoid isolated from licorice, modulates mitochondrial function and redox balance in leukemia cells. Chemoproteomic analyses have revealed that GLA covalently binds to the voltage-dependent anion channel 1 (VDAC1), thereby inducing mitochondria-dependent apoptosis associated with increased oxidative stress and mitochondrial damage [87]. In parallel, metabolic and stress-inducing agents such as metformin and homoharringtonine (HHT) exert cytotoxic effects through endoplasmic reticulum (ER) stress, inhibition of mitochondrial OXPHOS or complex I activity, and consequent redox imbalance, indirectly linking oxidative stress to DNA damage and apoptotic signaling in LSCs [88,89].

Several additional agents—including CR-LAAO (an oxidative enzyme derived from snake venom), TMQ0153 (a synthetic tetrahydrobenzimidazole derivative), ivermectin (an antiparasitic drug), and chlorogenic acid and gallic acid (a natural polyphenol)—promote intracellular ROS accumulation and oxidative stress in CML cells. This redox imbalance induces mitochondrial dysfunction and oxidative DNA damage, ultimately activating regulated cell death pathways [90,91,92,93,94]. ROS-mediated cytotoxicity is frequently amplified by NADPH oxidase activity (NOX1/NOX4) and mitochondrial respiratory inhibition, particularly when combined with TKIs [95,96]. The functional relevance of ROS is underscored by the ability of antioxidants to partially or fully reverse cytotoxic effects in most experimental systems [91,97].

Common therapeutic agents used in CML may induce DNA damage either independently of, or in parallel with, ROS generation. For instance, the synthetic retinoid ST1926 and the TKIs HS-543 and HS-438 promote apoptotic cell death in both imatinib-sensitive and -resistant CML cells, including those harboring the T315I mutation, through DNA fragmentation and activation of intrinsic apoptotic pathways [98,99,100]. Imatinib additionally inhibits topoisomerase I/II, contributing to ROS-independent DNA damage [101]. Notably, resistance is not always associated with defective DNA damage induction, as certain leukemic clones retain intact DNA repair signaling despite persistent genotoxic stress [76,102].

In addition, several structurally diverse compounds can exert therapeutic activity through concomitant activation of stress-responsive and mitochondria-dependent pathways. The HSP90 inhibitor SNX-2112 induces apoptosis in multidrug-resistant CML cells by suppressing Akt/NF-κB signaling and disrupting mitochondrial integrity [103]. Similarly, oroxylin A (ORM), a natural flavonoid, and tanshinone IIA, a diterpene quinone, trigger growth arrest and apoptosis through ERK- and JNK-mediated stress signaling, respectively [104,105]. YM155, a small-molecule survivin inhibitor, promotes autophagy-dependent downregulation of anti-apoptotic proteins and increases intracellular oxidative stress, whereas α-bisabolol, a natural sesquiterpene alcohol, induces mitochondrial dysfunction and ROS accumulation, collectively enhancing oxidative stress-driven cytotoxicity in CML models [106,107].

Finally, emerging drug delivery platforms based on nanoparticle systems co-encapsulating curcumin and paclitaxel offer the potential to mitigate oxidative DNA damage by improving tumor selectivity, enabling controlled drug release, and minimizing off-target oxidative stress, thereby improving therapeutic efficacy and safety profiles [108].

### 2.2. Mitochondrial Metabolic Reprogramming

**Figure 2:**
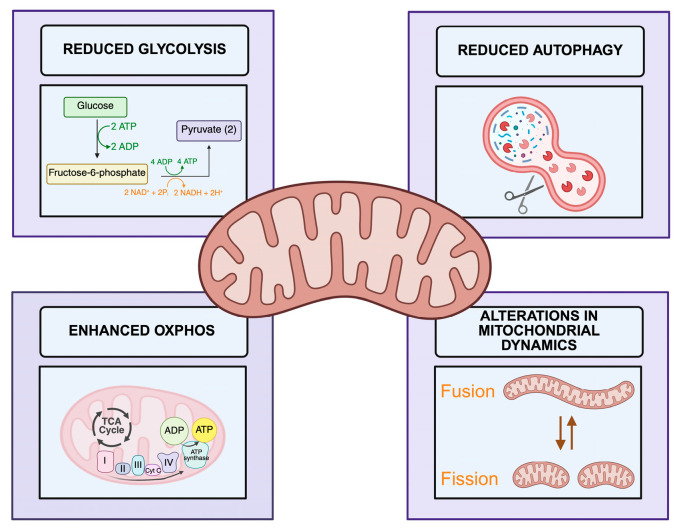
Altered Mitochondrial Metabolism in CML. This figure illustrates the key processes involved in mitochondrial metabolism in CML. Mitochondrial metabolism is reprogrammed in CML, characterized by downregulation of glycolysis and autophagy pathways, along with upregulation of oxidative phosphorylation (OXPHOS). This metabolic shift is accompanied by alterations in mitochondrial dynamics, including fission and fusion processes. Created with BioRender.com.

Mitochondria in cancer undergo extensive metabolic reprogramming to meet the anabolic and bioenergetic demands of uncontrolled proliferation. Although the Warburg effect traditionally describes a preferential reliance on aerobic glycolysis, it is now well established that many tumors retain active mitochondrial OXPHOS, particularly within cancer stem-like cell populations [109]. This metabolic adaptability allows cancer cells to flexibly exploit multiple nutrient sources, thereby sustaining tricarboxylic acid (TCA) cycle flux and ATP production [110]. A graphical overview of the effects of metabolic reprogramming in CML is provided in Figure 2.

#### 2.2.1. BCR-ABL-Mediated OXPHOS Dependence and Sensitivity to TKIs

In CML, mitochondrial metabolism is strongly shaped by oncogenic signaling. The BCR-ABL1 fusion kinase enhances mitochondrial biogenesis and oxygen consumption, particularly in LSCs, which exhibit reduced reliance on glycolysis and increased dependence on OXPHOS for survival and self-renewal [111]. Compared with normal hematopoietic stem cells, LSCs exhibit elevated OXPHOS activity, increased TCA cycle flux, and enhanced glucose oxidation, underscoring their distinct metabolic phenotype [23,88]. Notably, substantial metabolic heterogeneity exists within the LSC compartment: while some resistant populations maintain high OXPHOS activity, others exhibit suppressed respiration, impaired TCA cycle function, and reduced mitochondrial output, yet remain dependent on glycolytic enzymes such as pyruvate kinase M2 (PKM2) [64]. In certain settings, survival following TKI treatment may also rely on a metabolic shift toward glutamine-driven OXPHOS, and simultaneous inhibition of glycolysis and glutamine metabolism induces catastrophic energetic collapse and synergistic antileukemic effects [63].

Genetic alterations affecting metabolic enzymes—including isocitrate dehydrogenase (IDH), fumarate hydratase (FH), or succinate dehydrogenase (SDH)—lead to the accumulation of oncometabolites such as 2-hydroxyglutarate (2-HG), fumarate, and succinate. These metabolites inhibit α-ketoglutarate-dependent dioxygenases, driving epigenetic remodeling and pseudohypoxic signaling that contribute to malignant transformation and metabolic dysregulation [112]. Similarly, promoter hypermethylation-mediated downregulation of the mitochondrial ATP synthase β subunit (ATP5B) impairs OXPHOS and promotes metabolic adaptation associated with reduced drug sensitivity [113]. Alongside, a functional single-nucleotide polymorphism in FOLR3 alters folate metabolism, enhances mitochondrial activity, and increases clonogenic potential, correlating with decreased responsiveness to TKIs [72].

#### 2.2.2. Adaptive Metabolic Rewiring Under TKI Pressure

Although TKIs effectively suppress mitochondrial metabolism—affecting OXPHOS, ETC, glutaminolysis, and the TCA cycle—CML cells retain a remarkable capacity for metabolic adaptation. Under sustained therapeutic pressure, leukemic cells progressively restore mitochondrial function and remodel substrate utilization, a process driven in part by HIF-1, which promotes metabolic reprogramming and supports persistence [22,23].

Consistent with this notion, targeting HIF-1 signaling reverses OXPHOS suppression and reactivates mitochondrial metabolism, revealing potential vulnerabilities that can be exploited therapeutically. In this context, inhibition of the mitochondrial pyruvate carrier complex (MPC1/2), using compounds such as UK-5099, MSDC-0160, or the selective inhibitor 7ACC2, disrupts pyruvate import into mitochondria, uncoupling glycolysis from mitochondrial oxidation and destabilizing mitochondrial metabolism. These interventions exacerbate metabolic stress and increase vulnerability in TKI-treated cells [23].

Metabolic profiling of imatinib-resistant cells further supports the presence of broad metabolic remodeling, particularly affecting energy and lipid metabolism. These changes have prompted the identification of post-transcriptional regulators that contribute to this mitochondrial plasticity. Among them, miR-203a-5p-an epigenetically silenced tumor-suppressive miRNA targeting ABL1-is consistently downregulated in imatinib-resistant CML cells [114]. Loss of miR-203a-5p is associated with restored mitochondrial activity and altered substrate utilization, whereas its re-expression partially normalizes metabolic signatures [115].

Beyond substrate utilization, miRNA-mediated regulation also extends to mitochondrial structural dynamics. Comparative expression analyses of CD34^+^CD38^−^ LSCs from chronic-phase and blast-crisis CML patients revealed that loss of miR-142 promotes mitochondrial fusion and boosts respiratory function by depressing key metabolic and mitochondrial targets, including CPT1A, essential for fatty acid oxidation, and the fusion mediator MFN1. Restoration of miR-142 reverses these effects and increases sensitivity to therapy, underscoring the importance of miRNA-controlled mitochondrial dynamics in leukemic stem cell fitness [24].

Several agents, such as metformin, HHT, taxodione, and pyrvinium, may harness such abnormalities exerting antileukemic activity through modulation of mitochondrial metabolism [68,88,89,116]. In particular, HHT—a plant-derived alkaloid clinically used in hematologic malignancies—impaired mitochondrial complex I activity and OXPHOS, placing it within a broader class of mitochondrial-targeting agents with therapeutic relevance in CML [89].

In addition to direct modulation of metabolic enzymes and substrate flux, mitochondrial bioenergetics in CML can be influenced by calcium (Ca^2+^) homeostasis, a critical regulator of TCA cycle enzyme activity and OXPHOS. Lomerizine, a clinically approved voltage-gated calcium channel blocker, reduces cytosolic and mitochondrial Ca^2+^ availability in LSCs, thereby limiting activation of TCA cycle dehydrogenases, suppressing OXPHOS, and enhancing sensitivity to imatinib. These findings underscore Ca^2+^-dependent mitochondrial regulation as a therapeutically exploitable vulnerability in TKI-treated CML [117].

Mitochondrial metabolism in CML is tightly regulated by interconnected signaling pathways that coordinate mitochondrial biogenesis, redox balance, and energy sensing. The SIRT1–PGC-1α axis, a key regulator of mitochondrial biogenesis and oxidative metabolism, promotes OXPHOS and contributes to TKI resistance. Inhibition of SIRT1 or its downstream effector PGC-1α selectively reduces mitochondrial respiratory capacity without significantly affecting glycolysis, underscoring the pathway’s role in maintaining mitochondrial resilience [25].

Additional regulators refine mitochondrial output by coordinating glycolysis with oxidative metabolism. The kinase ULK1, beyond its role in autophagy initiation, modulates mitochondrial function and redox balance, while TXNIP acts as a metabolic checkpoint linking glucose availability to mitochondrial energy production. Together, these pathways fine-tune the balance between glycolysis and OXPHOS, reinforcing metabolic adaptability under therapeutic pressure [61,80].

#### 2.2.3. Mitochondrial Remodeling and Inflammatory Pathways

Direct pharmacological disruption of mitochondrial integrity—through targeting VDAC1, respiratory complex I, or mitochondrial membrane potential—represents an effective strategy to trigger bioenergetic collapse and cell death in leukemic cells [70,87,89,97]. Compounds including SNX-2112, gallic acid, compound 7b, and Ormeloxifene converge on mitochondrial dysfunction through complementary mechanisms, including impaired proteostasis, loss of membrane potential, and disorganization of electron transport chain complexes, ultimately resulting in ATP depletion and metabolic collapse [97,104,118].

Inflammatory signaling can further aggravate mitochondrial dysfunction by suppressing respiratory activity, depleting ATP, and increasing oxidative imbalance. Conversely, certain metabolic interventions display context-dependent protective effects; for example, inhibition of glycolysis can rebalance cellular energy metabolism, restore mitochondrial respiration, and reduce excessive ROS accumulation [74,75]. Additional agents such as YM155 and ivermectin directly localize to mitochondria, impair OXPHOS, inhibit cytoprotective autophagy, and promote oxidative stress, thereby limiting metabolic resilience and enhancing sensitivity to TKIs in CML models [81,92,106,119,120].

### 2.3. Mitochondrial Cell Death Pathways

#### 2.3.1. Canonical Intrinsic Apoptosis

Mitochondria are central effectors of the intrinsic apoptotic pathway and function as an integration hub for heterogeneous stress signals. Following cellular injury—such as growth factor deprivation, oncogenic stress, or genotoxic damage—pro-apoptotic BCL-2 family members (e.g., BAX and BAK) undergo conformational activation and oligomerize at the outer mitochondrial membrane. This process induces mitochondrial outer membrane permeabilization (MOMP), enabling cytochrome c release into the cytosol, apoptosome assembly, caspase-9 activation, and downstream executioner caspase engagement, ultimately culminating in programmed cell death [121]. In CML, this mitochondrial checkpoint is frequently restrained by overexpression of anti-apoptotic proteins (BCL-2, BCL-XL, MCL-1), which sequester BH3-only activators and preserve mitochondrial integrity, thereby sustaining leukemic survival and therapy resistance [121,122].

**Figure 3:**
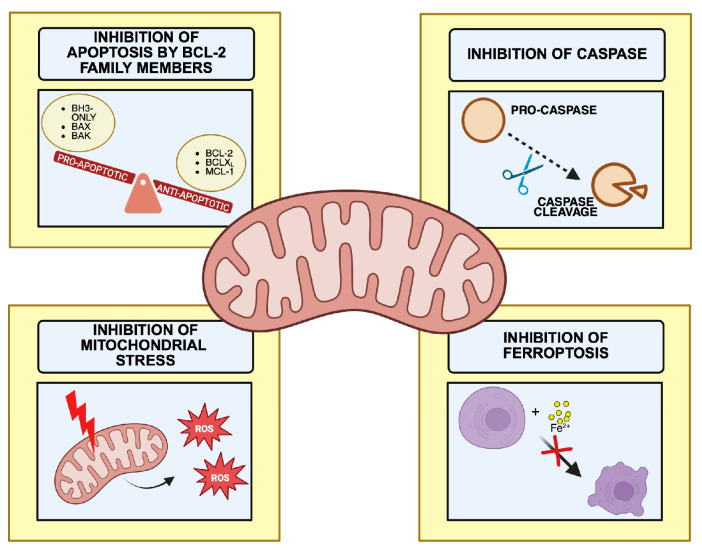
Mitochondrial regulation of cell death in CML. This figure illustrates the key processes involved in the regulation of cell death in CML. Cell death pathways are altered, with decreased levels of pro-apoptotic proteins, including the BH3-only proteins BIM, BID, PUMA, NOXA, BIK, and BAD, as well as the pore-forming proteins BAX and BAK, and increased levels of pro-survival proteins such as BCL-2, BCL-XL, and MCL-1. This imbalance results in impaired caspase activation and increased tolerance to mitochondrial stress, enabling the maintenance of mitochondrial integrity and bioenergetic capacity while preventing apoptotic signaling and promoting dysregulation of non-apoptotic cell death pathways. Created with BioRender.com.

Notably, canonical mitochondrial apoptosis is not uniformly engaged in all contexts, but becomes prominent under defined dual-stress conditions, like when glycolysis and OXPHOS are concomitantly suppressed (e.g., imatinib plus L-asparaginase or STAT3 inhibitors) [63,64]. A graphical overview of the alterations in cell death mechanisms in CML is presented in Figure 3.

#### 2.3.2. Pharmacological Induction of Mitochondrial Apoptosis

Numerous therapeutic strategies exploit mitochondrial pathways to induce apoptosis in CML. As anticipated above, many may converge on ROS accumulation, which affects mitochondrial integrity, reduces membrane potential, and triggers cytochrome c release with subsequent caspase activation—hallmarks of intrinsic apoptosis [81,93,101].

Radiation-based approaches indeed support mitochondria-centered cytotoxicity. Both low- and high-dose radiation decreases mitochondrial membrane potential, modulates caspase-3 activation, and shifts the BAX/BCL-XL ratio toward apoptosis [70]. Similarly, TKIs such as imatinib and nilotinib increase the pro-apoptotic BAX/BCL-XL ratio—primarily via BCL-XL downregulation—thereby promoting mitochondrial outer membrane permeabilization (MOMP) [123,124]. Furthermore, in combination with Nutlin-3, an MDM2 inhibitor, imatinib induces activation of BAX, promoting MOMP and subsequent caspase cascade activation [125].

Therapeutic interventions may engage mitochondrial apoptosis directly, by compromising membrane integrity, or indirectly, by altering BCL-2 family balance.

Additional compounds have also demonstrated antileukemic activity through mitochondrial-mediated mechanisms. These include dihydroartemisinin (DHA), a safe and effective antimalarial derivative of artemisinin that significantly suppresses BCR-ABL fusion gene expression at the mRNA level in CML cell [126]; the histone deacetylase inhibitor suberoylanilide hydroxamic acid (SAHA) combined with S116836, a novel multi-targeted tyrosine kinase inhibitor [127]; niclosamide, an FDA-approved anthelmintic effective against T315I BCR-ABL-expressing CML cells [128]; SNS-032, a cyclin-dependent kinase 7 (CDK7) and CDK9 inhibitor currently in phase I clinical trials [129]; and pyrvinium, another FDA-approved anthelmintic that selectively targets BP-CML CD34^+^ progenitor cells [116]. These agents exert their effects by promoting mitochondrial membrane destabilization, modulating BCL-2 family protein interactions, and/or inducing the release of apoptogenic factors such as AIF and cytochrome c.

Moreover, agents including GLA [87], metformin [88], HHT [89], TMQ0153 [91] and lomerizine [117], further induce mitochondrial dysfunction though through different mechanisms, including complex I inhibition, ATP depletion, and/or ROS accumulation, ultimately resulting in apoptotic cell death.

Disruption of stromal protection with the CXCR4 antagonist BKT140 promotes membrane depolarization and cytochrome c release, effects enhanced by imatinib [78]. Chloroquine instead activates intrinsic apoptosis through NOXA-dependent MCL-1 depletion and ERK inhibition, favoring pro-apoptotic BCL-2 signaling [95].

Oncogenic BCR-ABL may actively suppress mitochondrial apoptosis by preventing p53 translocation to the cytoplasm and mitochondria, thereby blocking Bax activation and its insertion into the outer mitochondrial membrane [76]. However, several strategies—including organopalladium compound 7b, ceramide combined with dasatinib, Tanshinone IIA, and dual-drug-loaded nanoparticles—may overcome this effect and restore mitochondrial apoptotic signaling [97,105,108,130].

Even in resistant settings, mitochondrial apoptosis can be reactivated. Ultraviolet exposure increases oxidative stress and DNA damage, enhancing mitochondrial depolarization and apoptosis in imatinib-resistant cells [71]. The BH3 mimetic ABT-737 lowers the apoptotic threshold—particularly in combination with TKIs—through membrane depolarization, caspase-3 activation, and HtrA2/Omi-mediated XIAP degradation [131]. Stress-adapted subsets such as FOLR3 SNP^+^ LSCs display mitochondrial overactivation and oxidative stress signatures linked to senescence-like phenotypes that may contribute to treatment-free remission [72,74].

Multiple SMs can trigger mitochondrial dysfunction and apoptosis despite distinct primary targets. These include gallic acid; HS-438; ST1926; MPT0B169; and α-bisabolol, all of which promote membrane depolarization, cytochrome c release, and caspase activation in CML cells [94,99,100,102,107]. Likewise, BIIB021 and ormeloxifen enhance apoptosis by remodeling BCL-2 family protein balance (upregulating Bak/Bad and downregulating Mcl-1), leading to caspase-9/-3 activation and PARP cleavage [104,132], emphasizing mitochondrial dysfunction as a key execution node.

Additional evidence links mitochondrial ROS to endothelial toxicity [79] and to apoptosis induction even in resistant contexts [68,75]. The small-molecule survivin inhibitor YM155 impairs mitochondrial integrity through downregulation of survivin and MCL1, thereby promoting apoptosis [106], while GATA4 upregulation enhances the expression of pro-apoptotic BCL-2 family members, shifting the balance toward programmed cell death [118].

#### 2.3.3. Metabolic Stress-Induced Apoptosis

Recent findings have further highlighted the pivotal role of mitochondrial function and metabolic reprogramming in the survival and elimination of CML stem cells, particularly under TKIs pressure. Inhibition of HIF-1 restores mitochondrial metabolism in otherwise quiescent LSCs, increasing their vulnerability to TKI-mediated clearance [22,23]. This combinatorial approach disrupts LSC quiescence, markedly reduces their long-term regenerative capacity, and drives functional exhaustion [22,23].

Beyond HIF-1-dependent pathways, additional mitochondrial regulators critically shape LSC fate through mitochondrial control. Loss of miR-142 leads to upregulation of the key mitochondrial GTPase MFN1, promoting mitochondrial fusion, which supports LSC survival by preventing mitochondrial fragmentation and consequent apoptosis, while its restoration reverses this phenotype [24]. Similarly, SIRT1 plays a crucial role in LSC maintenance. Genetic ablation of SIRT1 not only impairs leukemia progression but also markedly reduces LSC self-renewal; in combination with TKIs, SIRT1 deletion enhances apoptotic cell death through induction of mitochondrial stress [25].

Redox homeostasis and mitochondrial quality control further modulate death susceptibility. An altered NOX2/NOX4 balance disrupts the mitochondrial membrane potential and induces intracellular calcium overload, thereby activating cell death program [26]. TXNIP, as a negative regulator of the thioredoxin antioxidant system, exerts profound effects on mitochondrial integrity. TXNIP overexpression causes mitochondrial swelling, membrane depolarization, and heightened sensitivity to apoptosis, particularly evident in imatinib-resistant CML cells [61].

Disruption of mitochondrial architecture is also implicated in therapy-induced killing. Inhibition of DHODH induces marked alterations in mitochondrial morphology accompanied by loss of mitochondrial membrane potential, culminating in apoptosis rescued by uridine supplementation, reinforcing the centrality of mitochondrial pathways in DHODH-dependent lethality [66]. In this framework, some agents act primarily through upstream programs (e.g., metformin via endoplasmic reticulum stress [88]), whereas others engage mitochondria more directly (e.g., GLA and TMQ0153), promoting ROS-dependent mitochondrial impairment that culminate in programmed cell death [87,91].

Importantly, mitochondrial features are also closely associated with resistance phenotypes. Cells displaying reduced mitochondrial membrane potential are frequently less responsive to TKIs and other agents [62,113]. Conversely, combining TKIs with compounds that disrupt mitochondrial function—such as the ATP synthase inhibitor oligomycin-A [96], pesticides including the anthelmintic niclosamide [128], or inhibitors of the ERK signaling pathway [69]—results in a marked enhancement of apoptotic cell death.

Finally, impairment of autophagy—mediated in part by loss of ATG7, a core autophagy-related gene essential for mitochondrial quality control—leads to accumulation of ROS and synergizes with TKIs to promote leukemic progenitor cell death [65], underscoring the role of redox buffering and autophagic flux in maintaining mitochondrial integrity and contributing to therapeutic resistance.

#### 2.3.4. Induction of Non-Apoptotic Cell Death

Beyond apoptosis, mitochondria also participate in additional forms of regulated cell death, such as ferroptosis, an iron-dependent process driven by lethal lipid peroxidation. Ferroptosis is shaped by mitochondrial lipid metabolism and redox balance and is controlled by key regulators including acyl-CoA synthetase long-chain family member 4 (ACSL4), which promotes the incorporation of polyunsaturated fatty acids into membrane phospholipids, and GPX4, which suppresses ferroptosis by detoxifying lipid hydroperoxides [133]. Mitochondrial ROS and TCA cycle activity can further enhance lipid peroxidation and ferroptotic susceptibility [134,135].

Besides cell-autonomous killing, mitochondrial damage can also shape the immunogenicity of CML cell death. TMQ0153-induced necroptosis promotes the release of damage-associated molecular patterns (DAMPs), thereby increasing immunogenic output [91].

The synthetic methylated indolequinone MAC681 triggers mitochondrial cell death by rapidly depleting NAD^+^, dissipating the membrane potential, and causing mitochondrial calcium overload. These events lead to ATP collapse, mitochondrial swelling, and the release of AIF, culminating in caspase-independent necroptotic cell death that remains effective in TKI-resistant clones [136].

## 3. Conclusions and Future Directions

Thanks to the development and widespread use of BCR-ABL1–TKIs, CML has evolved from a fatal hematologic malignancy into a largely manageable chronic disease. Despite this remarkable clinical success, CML remains biologically complex and therapeutically challenging, particularly because of the persistence of LSCs likely driving the emergence of resistance in a subset of patients. Here, we summarize emerging evidence that mitochondria act as central regulators of CML pathobiology, integrating redox signaling, metabolic plasticity, and cell death control to sustain leukemic cell survival under oncogenic and therapeutic stress.

Accumulating evidence indicates that mitochondrial dysfunction in CML is not a mere consequence of transformation but an actively regulated process driven by BCR-ABL1 signaling and reinforced by epigenetic, transcriptional, and metabolic adaptations. LSCs, in particular, display a distinct mitochondrial phenotype characterized by increased reliance on OXPHOS, enhanced antioxidant defenses, and altered mitochondrial dynamics. These features enable LSCs to maintain quiescence, evade apoptosis, and survive prolonged TKI exposure, thereby constituting a reservoir for minimal residual disease and relapse.

Oxidative stress emerges as a double-edged sword in CML. At controlled levels, ROS function as signaling intermediates that promote proliferation, metabolic adaptation, and stem cell maintenance. When ROS exceed buffering capacity, however, they induce mitochondrial dysfunction, DNA damage, and activation of apoptotic or non-apoptotic cell death pathways. The fine balance between ROS production and antioxidant capacity therefore represents a critical vulnerability. Importantly, resistant CML cells often display enhanced redox buffering, underscoring the therapeutic potential of strategies that selectively disrupt redox homeostasis while sparing normal hematopoietic cells.

Metabolic reprogramming further reinforces mitochondrial resilience in CML. Rather than adhering strictly to the Warburg paradigm, leukemic cells—and especially LSCs—retain metabolic flexibility, dynamically switching between glycolysis, glutaminolysis, fatty acid oxidation, and OXPHOS in response to environmental cues and drug pressure. This plasticity limits the durability of single-agent metabolic interventions but simultaneously creates opportunities for rational combination strategies that collapse compensatory pathways. The emerging roles of HIF-1 signaling, SIRT1–PGC-1α-mediated mitochondrial biogenesis, calcium-dependent metabolic regulation, and miRNA-controlled mitochondrial dynamics emphasize that mitochondrial metabolism is tightly embedded within broader signaling networks.

Mitochondria also represent a convergence point for multiple regulated cell death modalities in CML, including intrinsic apoptosis, necroptosis, and ferroptosis. Although canonical mitochondrial apoptosis is frequently suppressed by BCR-ABL-mediated inhibition of p53 signaling and overexpression of anti-apoptotic BCL-2 family proteins, numerous studies demonstrate that mitochondrial death pathways can be reactivated under defined stress conditions or through combination therapies. Notably, mitochondrial-targeting agents can induce cell death even in TKI-resistant settings and, in some cases, independently of classical caspase activation, thereby bypassing key resistance mechanisms.

From a translational perspective, these findings argue strongly for a shift away from strategies that exclusively target BCR-ABL kinase activity toward approaches that exploit mitochondrial vulnerabilities. Combining TKIs with agents that perturb mitochondrial metabolism, redox balance, calcium homeostasis, or mitochondrial quality control holds promise for eliminating persistent LSCs and improving rates of durable treatment-free remission. A comprehensive list of the most relevant agents targeting oxidative stress, mitochondrial metabolism, and cell death pathways in CML, as discussed in this review, is provided in Table 1. Notably, given the essential role of mitochondria in normal hematopoietic stem cells and non-malignant tissues, therapeutic selectivity and potential toxicity of these compounds require careful evaluation prior to clinical translation.

Prospectively, future research should focus on (i) defining mitochondrial biomarkers that predict response, resistance, and suitability for treatment-free remission; (ii) resolving metabolic heterogeneity within the LSC compartment at single-cell level; and (iii) integrating mitochondrial-targeted therapies into rational, biomarker-guided combination regimens. Advances in drug delivery systems, including mitochondria-targeted compounds and nanoparticle-based formulations, may further enhance therapeutic precision.

In conclusion, mitochondria occupy a central and previously underappreciated role in CML biology, linking oncogenic signaling, metabolic adaptation, redox control, and cell death resistance. Targeting mitochondrial function thus represents a compelling and biologically grounded strategy to overcome residual disease, prevent relapse, and ultimately move closer to curative therapy for this malignancy.

## Figures and Tables

**Table 1:** Therapeutic Strategies Targeting Mitochondrial Metabolism, Oxidative Stress, and Cell Death in CML.

	Therapeutic Strategies	Primary Target/Pathway	Mitochondrial Effect	Therapeutic Outcome in CML	Reference(s)
**Natural** **Agents**	Taxodione; CR-LAAO	Mitochondrial ROS	ROS accumulation	Oxidative damage to nuclear DNA, apoptosis	[68,90]
	Homoharringtonine (HHT); Ivermectin	OXPHOS; ETC (Complex I)	Reduced respiration, ATP depletion	Apoptosis, LSC targeting	[89,120]
	Glaucocalyxin A; gallic acid	VDAC; membrane potential	Mitochondrial membrane depolarization	Cytochrome c release, apoptosis	[87,94]
	Oroxylin A	STAT3; ERK; JNK pathways	Mitochondrial membrane depolarization	Enhanced apoptosis	[64,104]
	α-Bisabolol	undetermined	Mitochondrial membrane depolarization	Activation of intrinsic apoptosis	[107]
	DHA (dihydroartemisinin)	Bcr/Abl fusion gene	Inhibition of mitochondrial respiratory capacity and decrease in ATP production	Apoptogenic factor release	[126]
**Synthetic** **Compounds/** **pharmacological agents**	Amsacrine	Mitochondrial ROS	ROS accumulation	Nuclear DNA damage, apoptosis	[69]
	miR-142 restoration	NRF2 pathway; thioredoxin system	Reduced ROS buffering	Increased oxidative stress-dependent death	[24,61]
	Dual NOX2–NOX4 inhibition	NOX2/NOX4 balance	Restored redox equilibrium	Enhanced mitochondrial dysfunction	[26]
	Metformin; Pyrvinium	OXPHOS; ETC (Complex I)	Reduced respiration, ATP depletion	Apoptosis, LSC targeting	[88,116]
	UK-5099; MSDC-0160; 7ACC2	Mitochondrial pyruvate carrier (MPC1/2)	Glycolysis–OXPHOS uncoupling	Metabolic stress, TKI sensitization	[23]
	TKI + glycolytic/glutamine inhibitors	Glycolysis + glutaminolysis	Energetic collapse	Leukemic cell death	[25,63]
	Lomerizine	Mitochondrial Ca^2+^ signaling	Suppressed TCA cycle enzyme activity	Enhanced imatinib efficacy	[117]
	Chloroquine; ATG7 loss	Inhibition of autophagy	Accumulation of damaged mitochondria	ROS buildup, apoptotic sensitization	[65,95]
	DHODH inhibitors	DHODH	Mitochondrial membrane depolarization, ROS increase	Nuclear DNA damage, apoptosis	[66]
	Organopalladium compound 7b	VDAC	Mitochondrial membrane depolarization	Cytochrome c release, apoptosis	[97]
	ABT-737; ceramide + dasatinib	Anti-apoptotic proteins	Lower apoptotic threshold	Activation of intrinsic apoptosis	[130,131]
	Imatinib + HIF-1 inhibition	BCR-ABL + mitochondrial stress	Exit from LSC quiescence	Functional LSC exhaustion	[22]
	STAT3 inhibitors + TKIs	STAT3; ERK	Mitochondrial membrane depolarization	Enhanced apoptosis	[64,104]
	TMQ0153; MAC681	Mitochondrial ROS; AIF	NAD^+^ depletion, Ca^2+^ overload	Caspase-independent cell death	[91,136]
	GPX4 inhibition; ACSL4 activation	Lipid peroxidation	Mitochondrial ROS-driven lipid damage	Ferroptosis	[133]
	Ionizing radiation; UV	DNA damage	Persistent oxidative stress	Apoptosis	[70]
	CXCR4 antagonist BKT140	Stromal protection	Mitochondrial depolarization	Sensitization to TKIs	[78]
	Curcumin + paclitaxel nanoparticles	Mitochondrial & oncogenic pathways	Controlled ROS and drug release	Improved efficacy, reduced toxicity	[108]
	ST1926	Nuclear DNA	DNA fragmentation	Activation of intrinsic apoptotic pathway	[99]
	SNX-2112; BIIB021	Hsp90; Akt/NF-kB signaling	Mitochondrial membrane depolarization; Oxidative stress	Activation of apoptosis, increases sensitivity to TKIs	[103,132]
	YM155	Survivin	Mitochondrial membrane depolarization; OXPHOS impairment; ROS accumulation	Autophagy and oxidative stress	[106,119]
	SAHA + S116836; SNS-032	TKs; CDK7/CDK9	Mitochondrial membrane depolarization	Apoptogenic factor release	[127,129]
	Oligomycin A	ATP synthase (Complex V)	Mitochondrial membrane depolarization	Apoptotic cell death and sensitization to TKIs	[96]
	Nutlin 3	MDM2	Mitochondrial membrane depolarization	Apoptosis	[125]
	MPT0B169	Tubulin/Microtubules	Mitochondrial membrane depolarization	Apoptosis	[102]
	Ormeloxifen	Estrogen receptor (ER)	Imbalance of BCL-2 family proteins	Mitochondrial apoptosis	[104]

## Data Availability

No new data were created or analyzed in this study. Data sharing is not applicable to this article.

## Abbreviations

The following abbreviations are used in this manuscript:

2-HG	2-hydroxyglutarate
2G-TKI	second-generation tyrosin kinase inhibitor
ACA	additional cytogenetic abnormalities
ACSL4	acyl-CoA synthetase long-chain family member 4
AE	adverse events
AIF	apoptosis-inducing factor
ALL	acute lymphoblastic leukemia
allo-SCT	allogeneic stem cell transplantation
ATP5B	ATP synthase β subunit
CCyR	complete cytogenetic response
CML	chronic myeloid leukemia
CQ	chloroquine
CSC	cancer stem-like cell
CXCR4	C-X-C chemokine receptor type 4
DAMP	damage-associated molecular pattern
DHODH	dihydroorotate dehydrogenase
DMR	deep molecular responses
ELN	European LeukemiaNet
ETC	electron transport chain
FH	fumarate hydratase
FFS	failure-free survival
FOLR3	folate receptor 3
GLA	Glaucocalyxin A
GPX	glutathione peroxidase
GSH	glutathione
HHT	homoharringtonine
HIF-1α	hypoxia-inducible factor-1
HSC	hematopoietic stem cells
IDH	isocitrate dehydrogenase
LDR	low-dose radiation
LSC	leukemic stem cell
MFN1	Mitofusin-1
MMR	major molecular responses
MnSOD	manganese superoxide dismutase
MOMP	mitochondrial outer membrane permeabilization
MPC1/2	mitochondrial pyruvate carrier complex
NGS	next-generation sequencing
NOX2	NADPH oxidase 2
NOX4	NADPH oxidase 4
NRF2	Nuclear factor erythroid 2-related factor 2
OXPHOS	oxidative phosphorylation
PKM2	pyruvate kinase M2
PRC2	Polycomb Repressive Complex 2
ROS	reactive oxygen species
SDH	succinate dehydrogenase
SIRT1	NAD-dependent protein deacetylase sirtuin-1
SNP	single-nucleotide polymorphism
STAT3	Signal Transducer and Activator of Transcription 3
TCA	tricarboxylic acid cycle
TFR	treatment-free remission
TKI	tyrosin kinase inhibitor
TXNIP	thioredoxin-interacting protein
VDAC1	voltage-dependent anion channel 1
